# The evolution of caste-biasing symbionts in the social hymenoptera

**DOI:** 10.1007/s00040-018-0638-3

**Published:** 2018-06-29

**Authors:** D. Treanor, T. Pamminger, W. O. H. Hughes

**Affiliations:** School of Life Sciences, University of Sussex, Falmer, Brighton, BN1 9QG UK

**Keywords:** Social insects, Symbiosis, Caste determination, *Wolbachia*

## Abstract

The separation of individuals into reproductive and worker castes is the defining feature of insect societies. However, caste determination is itself a complex phenomenon, dependent on interacting genetic and environmental factors. It has been suggested by some authors that widespread maternally transmitted symbionts such as *Wolbachia* may be selected to interfere with caste determination, whilst others have discounted this possibility on theoretical grounds. We argue that there are in fact three distinct evolutionary scenarios in which maternally transmitted symbionts might be selected to influence the process of caste determination in a social hymenopteran host. Each of these scenarios generate testable predictions which we outline here. Given the increasing recognition of the complexity and multi-faceted nature of caste determination in social insects, we argue that maternally transmitted symbionts should also be considered as possible factors influencing the development of social hymenopterans.

## Introduction

The defining feature of eusociality is reproductive division of labor (Oster and Wilson 1979; Crespi and Yanega 1995). Within a colony, individuals of different castes specialize in either reproduction (i.e., queens) or parental care and foraging (i.e., workers), and in many social insects, in particular the social hymenoptera, these behavioral castes also exhibit irreversible morphological differentiation (Wilson 1971). As a consequence, social insect colonies have to produce a mixture of new reproductives and workers from offspring of the same sex, and a major theme of research in social insect biology concerns the developmental processes underlying this. Until relatively recently, the prevailing view was that the development of totipotent larvae into either reproductives or workers was determined largely or entirely by environmental effects, but a number of studies have now shown effects of genotype on caste determination across a range of social insects (Anderson et al. 2008; Schwander et al. 2010). In some species these genetic effects are plastic, with individuals of certain genotypes displaying a moderate propensity towards developing into either reproductives or workers (Hughes and Boomsma 2007, 2008; Schwander and Keller 2008) whilst in other species, caste is determined almost entirely by genotype (Julian et al. 2002; Fournier et al. 2005; Darras et al. 2014; Kuhn et al. 2018).

Here, we discuss how natural selection may cause maternally transmitted symbionts (MTSs) to influence caste determination in the social hymenoptera. Heritable symbionts are extremely widespread in insects and vary enormously in terms of their effects on host organisms (Moran et al. 2008; Weinert et al. 2015). Some symbionts, such as *Buchnera* in aphids, are obligate mutualists of their hosts, providing essential metabolic functions (Shigenobu et al. 2000), whilst others enhance host fitness without being essential for survival (Oliver et al. 2003; Scarborough et al. 2005). Nevertheless, many MTSs have fundamentally selfish interests that differ from their hosts, because they can only be transmitted by females (Engelstädter and Hurst 2009a, b; Duron and Hurst 2013; Bennett and Moran 2015; Hurst and Frost 2015). This simple fact has led to the evolution of a profusion of manipulative traits in MTSs that increase the production of female offspring by infected hosts (Werren et al. 2008).

Heritable symbionts appear to be just as common in the social hymenoptera as they are in insects generally; numerous studies have reported the presence of complex symbiotic communities in ants, including taxa known to manipulate host reproduction (Wenseleers et al. 1998; Russell et al. 2009, 2012; Kautz et al. 2013), and such reproductive parasites have also been detected in social bees and wasps, as well as their solitary cousins (Evison et al. 2012; Gerth et al. 2013, 2015; de Oliveira et al. 2015). However, in the social hymenoptera, reproductive manipulation by MTSs may also extend to caste determination; only individuals of the reproductive caste are capable of propagating MTSs, and so these symbionts have a strong evolutionary interest in ensuring that first, they preferentially infect queens, and second, that their hosts preferentially produce queens.

### Three distinct selective pressures can lead to the evolution of caste-biasing by symbionts

Bourke and Ratnieks (1999) suggested that MTSs such as *Wolbachia* might be party to social conflicts concerning caste determination in insect societies. They argued that, because symbionts such as *Wolbachia* cannot be transmitted through sterile workers, symbionts should evolve to manipulate the development of females so that they are more likely to develop into queens. These queens will then be capable of transmitting the infection to the next generation. On the other hand, Wenseleers (2001) argued that, since all females in a colony are clonally related from the perspective of an MTS, the MTS will be selected to mutualistically maximize colony productivity rather than bias the caste fate of developing larvae.

Whilst this logic is sound, it rests on three important assumptions: (1) that the fidelity of transmission of the symbiont from parent to offspring is perfect, i.e., infected queens produce only infected offspring; (2) that only a single maternal lineage is present within a colony; (3) that any symbiont present has no interest in distorting the sex ratio of its host. In reality, each of these assumptions is violated in nature and each creates a different arena for conflict over caste determination between MTSs and social hymenopteran hosts. Here, we discuss each of these scenarios in turn and describe how they might be experimentally investigated.

#### Caste-biasing due to imperfect vertical transmission of symbionts

In many instances, the fidelity of transmission of MTSs to the offspring of their host is impressively high, and essentially all the offspring of an infected female are themselves infected (e.g., Shoemaker et al. 2003). Nonetheless, there are many cases in which the fidelity of transmission of MTSs is less than perfect; in other words, infected females produce some uninfected offspring (Hoffmann et al. 1990; Hurst et al. 2001; Wenseleers et al. 2002; Graham and Wilson 2012; Oliver et al. 2014; Dykstra et al. 2014), despite the fact that ensuring a high transmission rate is clearly in the evolutionary interests of MTSs. In solitary insects, MTSs will be under selection to maximize their transmission to the female offspring of their host, because only female offspring are capable of propagating the symbiont. However, in many of the social hymenoptera, only queens can reproduce, and workers are either completely sterile or only capable of producing male offspring. As such, in social hymenopterans, MTSs will be subject to selection to maximize the infection rate of queens, rather than females generally. We suggest that an MTS infecting a social hymenopteran could maximize the infection rate of queens by increasing the likelihood that infected female offspring develop into queens rather than workers. In a simple scenario, a caste-biasing MTS might alter larval begging or feeding behavior, lengthen the developmental period of larvae, or reduce the effect of queen pheromones that inhibit the development of female larvae into new queens.

In a more sophisticated scenario, the MTS itself could become an additional caste-determining locus, being necessary but not sufficient for the development of female larvae into reproductives. MTSs might interfere with caste determination at a molecular level, perhaps even becoming an essential part of signaling pathways that lead to the development of larvae into queens. Such fundamental effects of MTSs on host biology are not without precedent; in some lineages of the isopod crustacean *Armadillidium vulgare, Wolbachia* has become the sex-determining locus, supplanting nuclear sex determination (Cordaux et al. 2011). An alternative mechanism might involve a parallel with the Medea phenotype found in *Trilobium* beetles. When a female has a copy of the gene *medea*, any of her offspring that fail to inherit a copy die as zygotes. Thus, the *medea* gene ensures that all the offspring produced by a female contain copies of itself (Beeman et al. 1992). Werren and O’Neill (1997) suggested that MTSs are likely to cause similar phenotypes, in which uninfected offspring of infected hosts will die or suffer reduced fitness. An equivalent situation could occur with regard to the caste fate of larvae. Instead of an MTS causing mortality in offspring that do not inherit it, it would cause uninfected offspring to develop into workers instead of queens.

A clear prediction of this hypothesis is that in species with a caste-biasing MTS, the prevalence of infection will be lower in the worker caste than in queens. Interestingly, this pattern has been observed for *Wolbachia* infection in a number of social insect species (Keller et al. 2001; Van Borm et al. 2001; Wenseleers et al. 2002; Frost et al. 2010; Roy et al. 2015). In the past, this has been attributed to other causes—in particular the proposed adaptive loss of infection from the worker caste (Keller et al. 2001; Wenseleers et al. 2002). Just as an MTS may be selected to preferentially infect the queen caste, if they impose a fitness cost upon their hosts, it follows that they will be selected to reduce their transmission to sterile workers. In this case, the interests of the MTS and the host colony will be aligned, as the host will also benefit from the reduced burden of infection in the worker caste (Wenseleers et al. 2002). Other explanations for reduced infection rates in the worker caste include ovarial regression in workers, or age-dependent changes in symbiont titers (Keller et al. 2001; Wenseleers et al. 2002; Russell 2012), but such relationships could alternatively be explained by the presence of a caste-biasing MTS with imperfect vertical transmission. These alternative hypotheses could be distinguished by examining the relative infection rates of larvae, queens, and workers (see Table 1.) Furthermore, it has recently been shown that *Wolbachia* infection in *Aedes aegypti* mosquitoes can be determined non-lethally using near-infrared spectroscopy (Sikulu-Lord et al. 2016). If applied to social hymenopterans, this would allow the developmental fate of larvae of known infection status to be tracked, providing a means of directly testing for the presence of a caste-biasing MTS.

**Table 1:** Examining the relative infection rates of larvae, queens, and workers provides a means of testing for caste-biasing MTSs, and distinguishing these from other causes of relatively low MTS infection rates in workers

Larval infection rate	Queen infection rate	Worker infection rate	Explanation
$x$	$x$	$x$	No adaptive loss or caste-biasing
$x$	$x$	$<x$	Adaptive loss, ovarial regression or age-dependent changes in symbiont titer in the worker caste
$x$	$>x$	$< x\,{\text{or}}\, \sim x$	Caste-biasing MTS

#### Caste-biasing due to the coexistence of multiple maternal lineages

In insect societies, genetic conflict over queen rearing occurs under two circumstances (Ratnieks et al. 2006). Firstly, queens of some species mate with multiple males, and this creates potential conflict between patrilines over queen rearing, as genes which increase the propensity of larvae to develop into sexual offspring may spread despite the probable colony-level cost of over-producing new queens; accordingly, patrilines which are overrepresented in sexual offspring have been identified in the honeybee *Apis mellifera* and the ant *Acromyrmex echinatior* (Moritz et al. 2005; Hughes and Boomsma 2008). Secondly, the presence of multiple matrilines within a colony also generates conflict over queen rearing. There are numerous examples of social hymenopterans with polygynous colonies formed of unrelated queens (Stille and Stille 1992; Evans 1996; Carew et al. 1997; Heinze and Keller 2000; Brown et al. 2003; Hacker et al. 2005; Holzer et al. 2008; Helantera et al. 2013) and as a consequence, such colonies will consist of multiple matrilines. However, unlike with the presence of multiple patrilines within a colony, the presence of multiple matrilines will not only generate conflict between nuclear genes, but also between nuclear genes and symbiont genes. In a monogynous colony of a social hymenopteran, all offspring will share the same strain of any MTS that infects their mother. In this case, as noted by Wenseleers (2001), all individuals within such a colony are clonally related from the perspective of an MTS, so there should be no selection on MTSs to manipulate larval caste fate. The same reasoning also holds for secondarily polygynous colonies that readopt related queens. However, we suggest that the presence of multiple maternal lineages introduces the potential for intra-colony conflict between infected and uninfected lineages. Any strain that causes its host to preferentially produce queens will be at a selective advantage compared to queens that are uninfected or infected by a strain that does not bias caste determination; they will not have to pay the cost of producing workers, but will still be able to produce queens, and so will have a greater reproductive output.

This situation is not without precedent. At its simplest, a subset of queens in certain species appear to selfishly contribute more to the production of sexual offspring, and less to the production of new workers (Ross 1988; Fournier et al. 2004). In addition, alternative reproductive morphs, including varying degrees of queen dimorphism, are surprisingly common in ants (Heinze and Tsuji 1995; Heinze and Keller 2000). Whilst this might occur for a number of reasons, in at least some cases smaller morphs called microgynes behave as intraspecific social parasites of colonies also inhabited by larger queens called macrogynes (Wolf and Seppä 2016). For instance, in the ant *Myrmica rubra*, microgynes produce a much higher proportion of queens relative to macrogynes, essentially parasitizing the production of workers by macrogynes (Elmes 1976; Pearson and Child 1980; Leppänen 2012; Schär and Nash 2014). Furthermore, whilst some gene flow still occurs between the microgyne and macrogyne lineages, there is evidence of genetic divergence between the two (Leppänen et al. 2015, 2016). The social parasite *Mycocepurus castrator* has taken this a step further; it appears to have originated as an intraspecific social parasite of *M. goeldii*, but has subsequently become entirely reproductively isolated from its host, and the two are now considered to be distinct species (Rabeling et al. 2014).

It may even be the case that MTSs assist in the sympatric speciation of hosts and their social parasites, as well as being a causal factor in the initial selfish production of queens instead of workers. For instance, *Wolbachia-*induced mating incompatibilities contribute to reproductive isolation between *Drosophila recens* and its sister species *D. subquinaria*, acting in concert with behavioral isolation (Shoemaker et al. 1999; Jaenike et al. 2006). In *M. rubra*, the presence of a parasite queen generally prevents the production of males by host queens, but hosts do still produce males occasionally, and whilst these males appear less inclined to mate with parasite females, they occasionally do so (Leppänen et al. 2016). Infection with an MTS that also induces mating incompatibilities might explain how social parasites can genetically diverge from their hosts in sympatry, as appears to be the case in *M. rubra*, even when behavioral isolation is incomplete. MTSs could thus not only drive the initial evolution of selfish reproductive behavior, but also assist in the progression from intraspecific parasite to interspecific parasite. We suggest that alternative reproductive morphs, intraspecific social parasites, inquilines and their hosts should be screened for the presence of reproductive parasites to examine this possibility. Subsequent experiments combining antibiotic treatments, quantification of caste ratios and controlled mating between hosts and parasites could reveal whether social insect lineages are infected with caste-biasing MTSs.

#### Caste-biasing as a means of distorting host sex ratios

A number of sex-ratio-distorting MTSs are found across arthropods (Duron et al. 2008; Engelstädter and Hurst 2009a; Hurst and Frost 2015). Some cause the death of host males at an early embryonic stage, some feminize genetic males, and others induce parthenogenesis in their hosts, all of which lead to female-biased host sex ratios (Werren et al. 2008). These phenotypes have evolved because males are a reproductive dead-end from the perspective of maternally transmitted symbionts, so it is not surprising that they all involve reducing the number of males produced in order to increase the production of females. However, sex ratios in social hymenopterans, in which diploid eggs can develop into workers or queens, is determined as much by the proportion of diploid eggs that develop into queens rather than workers (i.e., the caste ratio) as it is by the relative proportion of haploid and diploid eggs. As discussed earlier, there is mounting evidence that such caste ratios can have a genotypic component (Anderson et al. 2008; Hughes and Boomsma 2008; Schwander et al. 2010).

In some species, genotypic effects on caste determination, and thus caste ratios, can go on to affect colony sex ratios in social insects. In the ant *Cardiocondyla kagutsuchi*, different genetic lines produce markedly different sex ratios; interestingly, this is due to differences between genetic lines in the likelihood of female larvae developing into reproductives rather than differences in the primary sex ratio between genetic lines (Frohschammer and Heinze 2009). MTSs in social hymenopterans could employ a very similar strategy, distorting host sex ratios by altering the caste fate of developing larvae such that female larvae are more likely to develop into queens than workers. Evidence for such an effect has recently been found in the ant *Monomorium pharaonis*. Experimental crosses between *Wolbachia*-infected and uninfected lineages have shown that infected colonies have more female-biased sex ratios than uninfected colonies and, whilst this is largely driven by reduced production of males in infected colonies, there also appeared to be a weaker effect of infection on caste ratio, with infected colonies producing more reproductive females relative to workers (Pontieri et al. 2016). Future tests of caste-biasing by MTSs as a means of distorting host sex ratios will require further comparisons of caste and sex ratios either in natural populations of mixed infection status, or in experimental populations in which infection status has been manipulated to allow caste and sex ratios to be compared whilst controlling for both host genetic background and environmental effects.

### Symbiont-induced thelytokous parthenogenesis and caste determination in the social hymenoptera

In some ant species, caste is determined entirely by the mode of reproduction; workers are produced via sexual reproduction, whilst queens are produced by thelytokous parthenogenesis (Fournier et al. 2005; Leniaud et al. 2012). At least three different MTSs are capable of inducing thelytokous parthenogenesis in arthropods (Ma and Schwander 2017), and so MTSs could, in theory, control caste determination by altering the mode of host reproduction. However, a number of obligately and facultatively parthenogenetic social hymenopterans have been screened for *Wolbachia* and other symbionts known to induce thelytokous parthenogenesis, and none appear to be infected (Grasso et al. 2000; Wenseleers and Billen 2000; Himler et al. 2009; Kronauer et al. 2012; Martinez-Rodriguez et al. 2013; Rabeling and Kronauer 2013). Initially, symbiont*-*induced parthenogenesis was thought to proceed only via gamete duplication, which eliminates genomic heterozygosity entirely (Ma and Schwander 2017). Because the majority of social hymenopterans utilize single-locus complimentary sex determination, gamete duplication would lead to the production of sterile diploid males instead of females, and so parthenogenesis-inducing symbionts were assumed to be unable to invade social hymenopterans (van Wilgenburg et al. 2006). However, it is now understood that a variety of cellular mechanisms underlie symbiont-induced parthenogenesis, some of which preserve heterozygosity (Ma and Schwander 2017) and so it is our opinion that the predominance of single-locus complimentary sex determination is not a sufficient explanation for the absence of symbiont-induced parthenogenesis in the social hymenoptera.

Alternatively, it has been proposed that symbiont-induced parthenogenesis is rare in the social hymenoptera for two related reasons, dependent on whether or not workers are sterile (Goudie and Oldroyd 2018). In species with sterile workers, thelytokous reproduction by queens would result in a loss of genetic variation in the worker caste, leaving the species more prone to extinction due to factors such as the accumulation of deleterious mutations and a reduced ability to adapt to fluctuating biotic and abiotic environmental factors (Maynard Smith 1978; Normark et al. 2003; Ross et al. 2013), as well as by removing the more specific benefits provided by a genetically variable worker caste, such as enhanced disease resistance and worker task specialization, rendering the species less competitive in relation to lineages that reproduce sexually (Wiernasz et al. 2008; Goudie and Oldroyd 2018). In species with workers that are not sterile, symbionts will likely cause parthenogenesis in workers as well as queens, precipitating a collapse in reproductive division of labor (Goudie and Oldroyd 2018). More generally, for thelytokous parthenogenesis to play a role in caste determination, it must be facultative, and with the exception of *Trichogramma* wasps, facultative sex has not been observed in species infected with parthenogenesis-inducing symbionts (Ma and Schwander 2017). In conclusion, MTSs are highly unlikely to affect caste determination in the social hymenoptera by inducing parthenogenesis in their hosts.

### Is caste-biasing likely to evolve in practice?

It could be argued that MTSs have only evolved a limited number of manipulative phenotypes, i.e., cytoplasmic incompatibility, male-killing, feminization and parthenogenesis induction, during the course of tens of millions of years of evolutionary history in association with an enormous number of arthropod hosts (Engelstädter and Hurst 2009a; Gerth et al. 2014); perhaps then, it is unparsimonious to propose another origin of a manipulative phenotype. However, it is important to note that the different manipulative phenotypes are, in reality, broad categories describing outcomes in the host, and the cellular and molecular mechanisms underlying reproductive manipulations vary enormously across hosts and MTS strains (Hurst and Frost 2015). For instance, symbiont-induced parthenogenesis proceeds through at least four different cellular mechanisms in arthropods (Ma and Schwander 2017) suggesting independent origins of the phenotype. Furthermore, even when the cellular mechanisms of a manipulative phenotype are the same, the underlying molecular mechanisms may vary, again due to independent origins of the manipulative phenotype (Ma et al. 2014). For example, symbiont-induced cytoplasmic incompatibility is known to occur in at least three distinct bacterial taxa (Engelstädter and Hurst 2009a; Takano et al. 2017). The cytological mechanisms of these mating incompatibilities have been studied in hosts infected with *Wolbachia* and *Cardinium*, and are remarkably similar regardless of which symbiont the host is infected with (Gebiola et al. 2017), but comparisons of the genomes of incompatibility-inducing *Wolbachia* and *Cardinium* provide no evidence for shared genes underlying the induction of mating incompatibilities; cytoplasmic incompatibility thus appears to have (at the very least) two independent evolutionary origins, rather than a single origin followed by horizontal gene transfer between taxa (Penz et al. 2012). We argue that the limited number of categories of manipulative phenotypes are thus likely to represent many independent origins of manipulative phenotypes, and should not be taken to suggest that MTSs have limited evolutionary potential. In fact, quite the opposite appears to be the case; when the selective conditions are appropriate, MTSs appear readily able to evolve the ability to manipulate host biology through a range of different cellular and molecular mechanisms.

## Conclusions

Caste determination in social hymenopterans is increasingly recognized as a complex phenomenon, with multiple interacting causes. We suggest that heritable symbionts should also be considered as additional factors that may influence caste determination, because there are at least three distinct evolutionary scenarios that could select for caste-biasing genes in MTSs. Such symbionts are extremely widespread, easy to detect using a range of standard laboratory techniques, and all three evolutionary scenarios outlined here generate testable predictions concerning caste determination in social hymenopterans. Maternally transmitted symbionts are already renowned for their ability to influence fundamental aspects of metabolism, immunity, behavior and reproduction in their hosts and we suspect that caste determination will be no exception to this.
